# A multi-site service evaluation of silver diamine fluoride use for children

**DOI:** 10.1038/s41415-023-6175-0

**Published:** 2023-08-25

**Authors:** Laura Timms, Sara Bux, Linzi Maybin, Helen Rogers, Katie Horisk, Jacqueline Fraser, Jessica Large, Chris Deery, Paul Ashley, Alex Keightley, Oliver Sumner

**Affiliations:** 032599131388525837093 Doctoral Fellow, Paediatric Dentistry, University of Sheffield, United Kingdom; 231019963075183444433 Dental Officer, Central London Community Health, United Kingdom; 534876992475353092671 Senior Dental Officer, Bradford District Care NHS Foundation Trust, United Kingdom; 756299259094881700393 Clinical Lecturer, Paediatric Dentistry, University of Newcastle, United Kingdom; 086904656720747084714 Paediatric Dentistry Registrar, Eastman Dental Hospital, United Kingdom; 046531459279065538406 Dental Core Trainee 2 in Oral and Maxillofacial Surgery, Crosshouse Hospital, United Kingdom; 903781962109341210102 Paediatric Dentistry Registrar, Charles Clifford Dental Hospital, Sheffield, United Kingdom; 870047734095337475755 Professor in Paediatric Dentistry, University of Sheffield, United Kingdom; 612123514214499402208 Professor in Paediatric Dentistry, University College London, United Kingdom; 240193053850891691821 Consultant in Paediatric Dentistry, Edinburgh Dental Institute, United Kingdom; 893974062946341214707 Consultant in Paediatric Dentistry, Newcastle Dental Hospital, United Kingdom

## Abstract

**Introduction **The use of silver diamine fluoride (SDF) is relatively new to the UK. It is unknown how it is being used and for what indications in UK paediatric dental services.

**Aim **To: 1) establish how SDF is being used across different paediatric dental settings in the UK; and 2) consider parental and patient views on the treatment experience and side effect of discolouration.

**Method** A multi-site service evaluation was carried out across six paediatric dentistry units covering hospital and community services. Data were collected prospectively from 17/02/2020 to 02/03/2022. Simple descriptive statistics were used to analyse the data.

**Results **Data were collected for 54 patients. The included patients had an age range of 2-13 years, with a mean of 4.9 years. The reason SDF was chosen was reported as: to avoid general anaesthetic (n = 25); to avoid extractions (n = 8); stabilisation (n = 25); acclimatisation (n = 24); and insufficient cooperation for other treatment (n = 17). In total, 42 cases had SDF applied to the primary dentition. This was in the anterior dentition for 18 patients and the posterior dentition for 15, with nine patients having SDF applied both anteriorly and posteriorly. The majority of children and parents were accepting of the technique and immediate aesthetic outcome.

**Conclusion** In the services involved in this multi-site service evaluation, SDF is used for young patients in the primary dentition for the purpose of caries arrest. The technique was viewed positively by the majority of parents and children.

## Key points

- Silver diamine fluoride is used mostly for caries arrest in the primary dentition in the services represented in this project.
- The majority of parents and children find the technique acceptable.
- The majority of parents and children find the black discolouration of caries acceptable.

## Introduction

Silver diamine fluoride (SDF) is effective in arresting caries in primary teeth.^1^^,^^2^ It was licenced for use off-label for caries arrest in the UK when granted a CE mark for use in the European Union in 2017. Despite widespread use internationally, there was limited use in the UK. During the COVID-19 pandemic, the introduction of SDF into standard operating procedures from the British Society of Paediatric Dentistry and the Office of the Chief Dental Officer in England, helped increase its use.^3^^,^^4^ To our knowledge, there are no data regarding its use in the UK. Given its fairly recent introduction into practice, it is important to assess how SDF is being used.

**Fig. 1:**
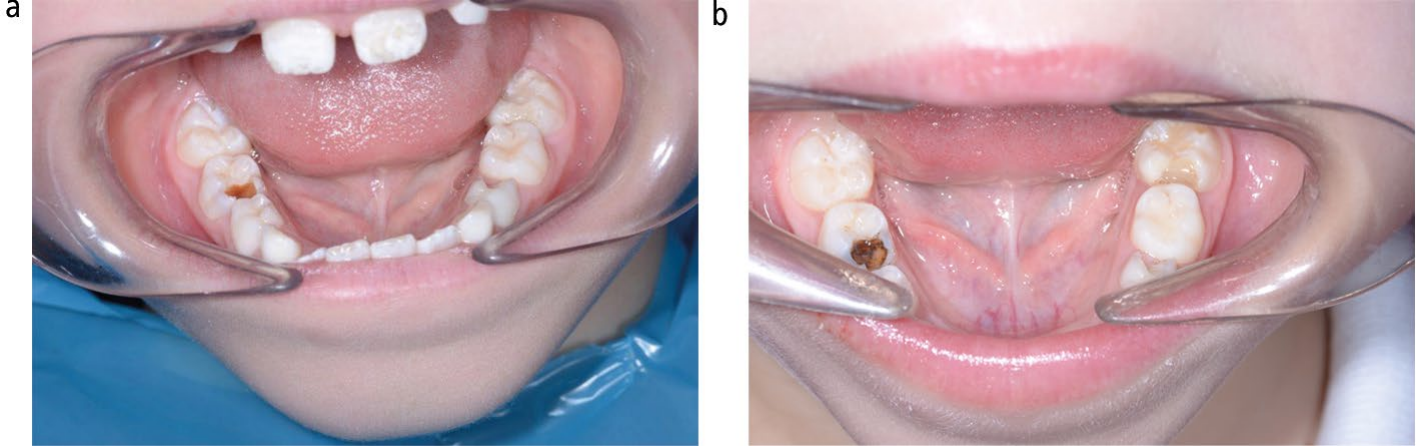
a) Pre-operative photograph of mesial-occlusal caries in the 85. b) Post-operative photograph of mesial-occlusal caries in the 85 following SDF treatment. Reproduced from Timms *et al*., 'Everyone else is using it, so why isn't the UK? Silver diamine fluoride for children and young people', vol 37, 2020, *Community Dental Health Journal*

SDF has a side effect of discolouring carious tooth tissue black, demonstrated in Figure 1. Internationally, acceptability from parents of this discolouration is varied, and is reported to be between 0% in studies in Saudi Arabia and Brazil to 100% in a study in Brazil.^5^ It is also important to consider patient and parent opinion of this new technique with a side effect that could potentially be a barrier to use.

## Aim

1. Establish how SDF is being used across different paediatric dental settings in the UK
2. Consider parental and patient views on the treatment experience and the side effect of discolouration.

## Methods

The project was initially started in two paediatric dentistry specialist units and subsequently other departments expressed interest in taking part. There were expressions of interest from nine units and of which, six units collected data. The participating sites were community dental services in Bradford, London and Edinburgh, and paediatric dentistry hospital dental services in Sheffield, Newcastle, Edinburgh and London (Eastman Dental Hospital).

Data were collected between 17/02/2020 and 02/03/2022. Data were collected at various time periods across different units, with some starting data collection at a later point than others. Data collection was limited in 2020 owing to the COVID-19 pandemic and the cessation of routine dental care.

Units were invited to prospectively complete a *pro forma* for all paediatric patient attendances that had application of SDF. Data collection included: patient demographics; reason and indication for SDF application; whether the activity status of the caries was arrested; the role of SDF in the treatment plan; and parental and child views of the treatment experience and side effect of discolouration. This was also collected at follow-up appointments.

Using a five-point scale of 'very happy' to 'very sad' faces, children were asked to rate their satisfaction with aesthetics and the treatment experience. Parents were also asked on a five-point scale the same aspects numbered 1-5 from 'very dissatisfied' to 'very satisfied'. These questions were asked after the first treatment and at subsequent follow-up visits.

Simple descriptive statistics were used to present the data. For analysis, timeframes were rounded to the nearest week, and one month was considered as four weeks.

Each unit followed their own local protocols regarding registration as a clinical governance project.

## Results

Data were collected for 54 patients across six different paediatric services. There were elements of missing data for some of the participants. The missing data were not consistent across all participants or sites.

The included patients had an age range of 2-13 years old, with a mean of 4.9 years old. Age-related data were not provided for three of the 54 patients. Data were available for the medical history of 49 patients, 33 of which were fit and healthy. Conditions that were relevant in patients' medical histories included cardiac, respiratory, haematological, neurological, craniofacial, gastric, metabolic and skin conditions, along with a range of syndromes.

Regarding the socioeconomic status of the patients included, 32.0% (n = 14) and 23.3% (n = 10) were from the most deprived and second most deprived deciles in the country, respectively. As such, more than half the patients were from the most deprived quintile in England (data from Edinburgh was not provided for this factor).

There were missing data from all units, particularly the hospital services. A particular area where there was paucity in data collection was follow-up appointments. Data were recorded at the first treatment visit for 54 patients and for 25 children at a follow-up visit, with three not being brought to planned follow-ups. Data were available for a second follow-up visit for nine patients and one was not brought to their second follow-up. The range to follow-up visit one was 2-24 weeks, with a mean of 6.8 weeks. Following this, the range to follow-up visit two was 8-52 weeks, with a mean of 18.75 weeks.

There were data for decayed, missing and filled primary teeth for 51 patients of the 54 with the mean number being 7.9, and the range being from 1-20.

In regard to decayed, missing and filled teeth in the permanent dentition, the quality of the data was poor; it was not recorded for 28 children, while 'not applicable' was recorded for 14 where there were no permanent teeth erupted, with data available for 12 children. Of the 12 patients with data available in the permanent dentition, the range was 0-5, with 1.25 being the mean.

In regard to treatment planning with SDF, 20 patients had SDF solely applied and 31 had SDF applied in conjunction with another treatment option. These were: SDF, preformed metal crowns and extractions (n = 6); SDF and fluoride varnish applied elsewhere (n = 9); SDF and a temporary restoration (n = 4); and SDF and general anaesthesia (n = 1). Additional treatment was not specified for nine cases. The treatment planning aim when using SDF application was recorded for 44 of the patients. This was for the indication of caries arrest alone (n = 28) and caries arrest and prevention (n = 15). In one instance, SDF application was for the management of sensitivity associated with molar incisor hypomineralisation.

The reason SDF was chosen rather than alternative treatments was reported as: a wish to avoid general anaesthetic (GA) (n = 25); a wish to avoid extractions (n = 8); stabilisation (n = 25); acclimatisation (n = 24); and insufficient cooperation for other treatment (n = 17). Where there was a wish to avoid extractions, this was for a medical reason in one case, and to avoid anterior extractions in two patients; the remainder did not specify why this was the preference. Other reasons reported for SDF choice for individual patients were: to avoid local anaesthesia and conservative management; molar incisor hypomineralisation sensitivity; to prevent a more invasive treatment; minimal caries with teeth close to exfoliation; to avoid the use of preformed metal crowns due to aesthetics; and stabilisation of other teeth while awaiting GA for extractions due to a long waiting list in order to prevent other teeth becoming symptomatic.

For 44 patients where data were available, 42 cases had SDF applied to the primary dentition. This was in the anterior dentition (primary incisor or canine) for 18 patients, the posterior dentition (primary molars) for 15, and nine patients had SDF both anteriorly and posteriorly.

Two patients had SDF applied in the permanent dentition to their first permanent molars.

When patients had a follow-up, it was reported whether caries had arrested. There was follow-up data for 22 patients related to the success of treatment for their subsequent visit, in which caries was reported as arrested for 14 patients, four had partial arrest of the caries, three had successful arrest for some teeth and not others, and one dentist reported they could not assess the success of treatment. At the second follow-up, there were data for only eight patients for this parameter; for five this was successful and for three there was caries arrest for some teeth but not others.

Regarding each child's experience, 21 (44.7%) found the procedure 'very easy' and eight (17.0%) found it 'easy'. Eight (17.0%) reported that they 'didn't mind' and nine were 'unhappy' (19.1%), with one 'very unhappy' (2.1%). On some forms, it was reported that some patients did not understand the question and as such, no data were given. These data were from the first treatment visit. At follow-up visits, ten children were 'very happy' with the procedure (66.7%), one was 'happy' (6.7%), three 'didn't mind' (20%), and one was 'unhappy' (6.7%). There was only data for four patients that had reapplication on their second follow-up. Of these patients, three were 'very happy' and one 'did not mind'.

**Fig. 2:**
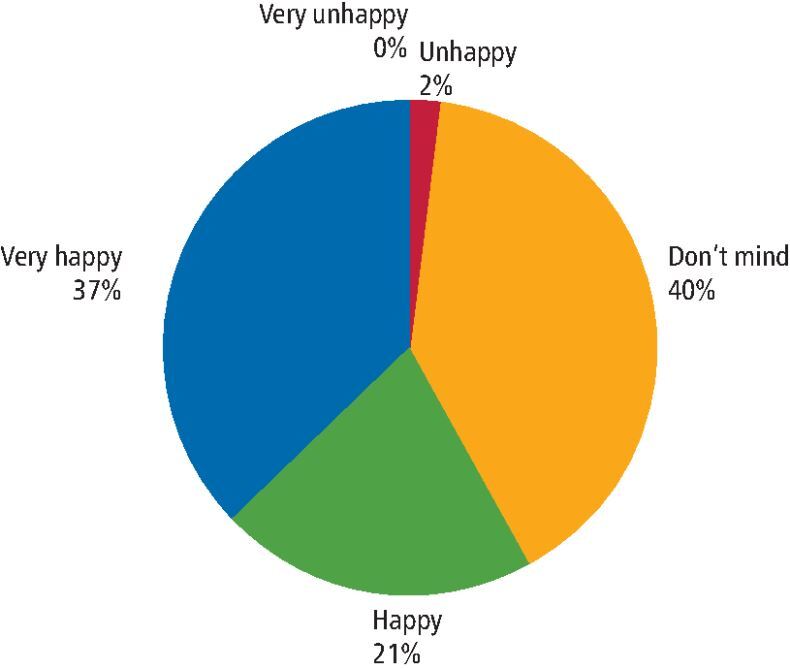
Pie chart demonstrating child satisfaction with aesthetics following SDF treatment at visit one

Children were asked to report on their view of the aesthetics of the treatment at the treatment visit. This is reported in the pie chart in Figure 2. These were data for the first treatment visit.

At the second follow-up, in cases where SDF treatment was reapplied, seven patients were 'very happy' (53.8%) and six were 'unhappy' (46.2%). There were data for four patients' third follow-up, where two were 'very happy', one was 'happy' and one 'did not mind' the aesthetics.

Parental views were also reported. At the first visit, the majority were happy with the procedure: 33 (63.5%) were 'very happy', 18 (34.6%) were 'happy' and one (2%) 'did not mind' it. The second follow-up visit had similar results, with 14 (82.3%) being 'very happy' and three (17.6%) 'happy'; no other results were reported. The third follow-up had limited data; of five parents, four were 'very happy' (80%) one was 'happy' (20%).

**Fig. 3:**
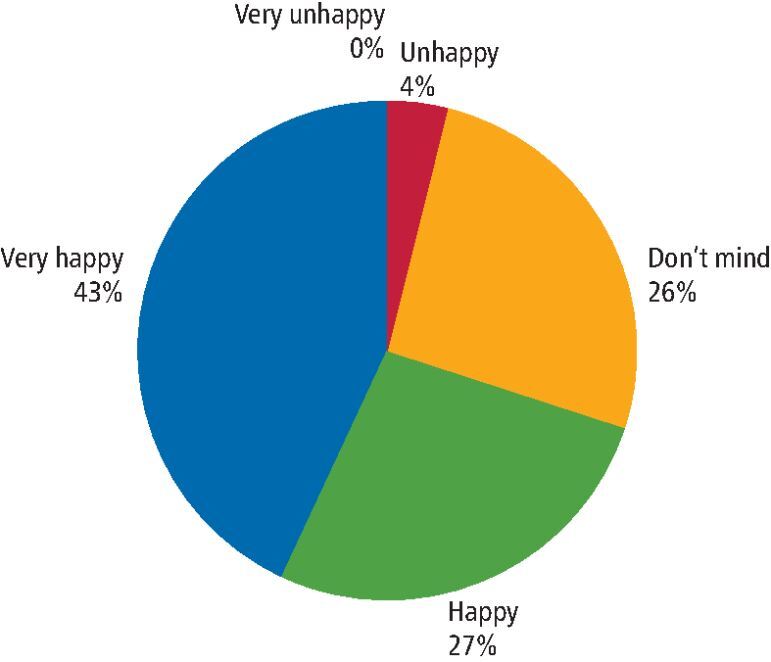
Pie chart demonstrating parental satisfaction with aesthetics immediately following SDF treatment

The parents' view on aesthetics at the end of the visit after the first treatment application is demonstrated in Figure 3.

At the second follow-up, ten (58.8%) parents were 'very happy', three were 'happy' (17.6%), two 'didn't mind' (11.8%) and two were 'unhappy' (11.8%). The third follow-up only had data from five parents, where four were 'very happy' (80%) one was 'happy' (20%).

Comments from parents and children were recorded. These were given by 23 patient/parent pairs. The majority were positive:

- 'Easy session and happy'
- 'Happy with treatment'
- 'Quick and easy'
- 'Don't mind as long as it works'
- 'Happy because it avoids extractions at such a young age'.

The more negative comments were:

- 'Child upset as did not like the taste'
- 'Agreed not to do front teeth'
- 'Hopefully can create a product that reduces blackness'.

## Discussion

This project provides an indication as to how SDF is being used on paediatric patients in specialist hospitals and community dental services. The majority of SDF use is for caries arrest in the primary dentition. This is in keeping with the body of the evidence base and reflects national standard operating procedures from the Chief Dental Officer and the British Society of Paediatric Dentistry.^3^^,^^4^ It is also in keeping with qualitative evidence from paediatric dentists in the UK, citing this as the indication in which they would use SDF.^6^ Appropriate evidence is the cornerstone of evidence-based dentistry and pivotal for decision-making in treatment planning for dentists in the primary dentition. As such, it is not surprising this is the most common use of SDF based on the available evidence.

The data show the use is common in younger children with high caries rates. This is again unsurprising given some of the benefits of SDF treatment, such as its ease of use for the child and its ability to treat many teeth in one visit. Further comments were reflective of other data around ease of use and families being accepting of the staining if the treatment is effective.^7^^,^^8^

The experience data further demonstrates that SDF is an easy procedure to accept. The high acceptance rates of the aesthetics from parents are unsurprising given these were all parents that consented to the treatment knowing this side effect but is nevertheless reassuring. It is important to note that the first treatment visit data asked about the aesthetics immediately following application and that the colour change is further established over time.

This was a service evaluation; clinicians were not calibrated and there was no recruitment strategy. Therefore, arrest rates cannot be treated as research evidence. However, despite this, it is interesting that the clinician-reported arrest rates were in keeping with success rates of SDF from systematic reviews.^1^^,^^2^

A limitation of the project is that it was originally designed by two services to fit their working practices. As such, when other services joined later, the data collection methods may not have suited their systems and working practices, which may have caused difficulties in data collection, resulting in some missing data, particularly at second and third follow-ups. There were fewer missing data from the community dental services. This may be attributed to continuity of care in these services, whereas, in the hospital, follow-up appointments are likely to be delivered by different clinicians. Regarding data for the permanent dentition, given the age profile of the participants, the majority with missing data are likely to have had no permanent teeth yet erupted.

Owing to the data collection period, some services collected data early, before SDF was recommended as an option by either the Chief Dental Officer or the British Society of Paediatric Dentistry, although the vast majority of data collection was following this. The COVID-19 pandemic and changes to practice as a result of this were during the data collection period, which may have affected clinicians' treatment planning during this time. Nonetheless, this is novel and useful data and interestingly, the utilisation of SDF for caries arrest in the primary dentition appears consistent throughout the time period of data collection.

A strength of this service evaluation was that it provides new data on the use of SDF from across the country. This can be further built on through research evidence on the implementation and effectiveness of SDF when utilised in the UK, both in secondary and primary care. There was some use for SDF in the permanent dentition in this service evaluation, albeit for only two patients. Given there is a more limited evidence base for this indication, it would be a clinically necessary area for further research investigation.

## Conclusion

In the specialist services in dental hospitals and in the community dental services involved in this multi-site service evaluation, SDF was used for young patients in the primary dentition for the purpose of caries arrest. The majority of patients and parents were accepting of the discolouration side effect and the treatment experience.

## References

[1] Seifo N, Cassie H, Radford J R, Innes N P. Silver diamine fluoride for managing carious lesions: an umbrella review. *BMC Oral Health* 2019; **19:** 145. 10.1186/s12903-019-0830-5 PMC6626340 31299955

[2] Trieu A, Mohamed A, Lynch E. Silver diamine fluoride versus sodium fluoride for arresting dentine caries in children: a systematic review and meta-analysis. *Sci Rep* 2019; **9:** 2115. 10.1038/s41598-019-38569-9 PMC6376061 30765785

[3] Office of the Chief Dental Officer. Standard Operating Procedure: Transition to Recovery. 2020. Available at https://www.england.nhs.uk/coronavirus/wp-content/uploads/sites/52/2020/06/C0575-dental-transition-to-recovery-SOP-4June.pdf (accessed July 2023).

[4] British Society of Paediatric Dentistry. Standard Operating Procedure for the Application of Silver Diamine Fluoride. 2020. Available at https://www.bspd.co.uk/Professionals/Resources (accessed July 2023).

[5] Sabbagh H, Othman M, Khogeer L, Al-Harbi H, Al Harthi A, Abdulgader A A. Parental acceptance of silver Diamine fluoride application on primary dentition: a systematic review and meta-analysis. *BMC Oral Health* 2020; **20:** 227. 10.1186/s12903-020-01195-3 PMC7439720 32819333

[6] Timms L, Graham, A, Gallacher N *et al.* Paediatric dentists' views on the use of silver diammine fluoride: a UK perspective. *Faculty Dent J* 2021; **12:** 114-119.

[7] Kyoon-Achan G, Schroth R J, Martin H *et al.* Parents' Views on Silver Diamine Fluoride to Manage Early Childhood Caries. *JDR Clin Trans Res* 2021; **6:** 251-257. 10.1177/2380084420930690 32479240

[8] Seifo N, Cassie H, Radford J R, Innes N P. 'I guess it looks worse to me, it doesn't look like there's been a problem solved but obviously there is': a qualitative exploration of children's and their parents' views of silver diamine fluoride for the management of carious lesions in children. *BMC Oral Health* 2021; **21:** 367. 10.1186/s12903-021-01730-w PMC8298692 34301214

